# Bacilli in the Air and Infection

**Published:** 1899-09

**Authors:** 


### Bacilli in the Air and Infection.

Those of us who, now quite a number of years ago, felt compelled to operate in the shower sent forth by the steam spray, experienced quite a sense of relief when it was discovered that it was an unnecessary measure. Theoretically it was useful, but in practice it was found that aseptic results could be obtained quite as well without it. Now Friedrich, of Leipsic, shows that the present methods are well justified. He recently stated that the danger of infection from bacilli floating in the air was generally overestimated, that they required a period of, at least, eight hours to take on their reproductive functions, while the tissues themselves, reacting almost immediately, were able to take care of those which might fall upon and contaminate the wound. According to him bacteria enter the circulation only under influence of pressure.

The last statement is one which may not be accepted without certain reservations, but there is no question that, given clean hands, clean instruments and aseptic solutions, the danger from bacteria contained in the air is exceedingly small. Of paramount importance is the vitality of tissue, without which the microbicidal reactions may not take place. Hence, it should ever be the object of the surgeon to impair this vitality as little as possible. Hemostatic forceps, by bruising and crushing the parts, are an evil, though a necessary one, and the surgeon should use as few as possible. He should also avoid placing too many ligatures, the most absorbent of which are at least for a time foreign bodies, which, by constricting the tissues, produce local devitalizations, favorable to the growth of germs. We can hardly escape the conclusion that pathogenic bacteria invariably fall upon the wounds we make, and that we can make wounds in which they will be harmless.—*International Journal of Surgery.*

